# Deep Learning for Historical Document Analysis and Recognition—A Survey

**DOI:** 10.3390/jimaging6100110

**Published:** 2020-10-16

**Authors:** Francesco Lombardi, Simone Marinai

**Affiliations:** Department of Information Engineering (DINFO), School of Engineering, Università degli Studi di Firenze, 50139 Florence, Italy

**Keywords:** artificial neural networks, deep learning, document image analysis and recognition, historical documents

## Abstract

Nowadays, deep learning methods are employed in a broad range of research fields. The analysis and recognition of historical documents, as we survey in this work, is not an exception. Our study analyzes the papers published in the last few years on this topic from different perspectives: we first provide a pragmatic definition of historical documents from the point of view of the research in the area, then we look at the various sub-tasks addressed in this research. Guided by these tasks, we go through the different input-output relations that are expected from the used deep learning approaches and therefore we accordingly describe the most used models. We also discuss research datasets published in the field and their applications. This analysis shows that the latest research is a leap forward since it is not the simple use of recently proposed algorithms to previous problems, but novel tasks and novel applications of state of the art methods are now considered. Rather than just providing a conclusive picture of the current research in the topic we lastly suggest some potential future trends that can represent a stimulus for innovative research directions.

## 1. Introduction

The field of Document Image Analysis and Recognition (DIAR) deals with the analysis and recognition of documents, traditionally focusing on scanned (imaged) documents [1]. In the earlier years, the research was mostly focused on text recognition initially dealing with printed text (OCR systems) and then with handwritten text. Successful applications, in the early 1990’s, were deployed in industrial systems, especially in the field of postal automation. The DIAR research area has been closely interlinked with techniques based on Artificial Neural Networks (ANN) [2]. The second wave of wide use of ANNs was significantly fueled by the introduction of the Backpropagation algorithm proposed by Rumelhart, Hinton, and Williams in 1986 [3] that allowed to train ANNs with nonlinear activation functions capable of a better representation of trainable functions than previous networks based on the Perceptron. In these years some sub-problems in DIAR, most notably the recognition of isolated handwritten digits, were instrumental for the study and development of more powerful learning paradigms thanks to the well defined application domain and to the availability of relatively large training datasets (like the MNIST [4]). This research reached a milestone at the end of the 1990’s with the proposal by Lecun, Bottou, Bengio, and Haffner [4] of the first widely recognized architecture based on Convolutional Neural Networks (CNN) that eventually lead to the third wave of research on ANNs that is still taking place today.

As previously noticed, initial uses of ANN on DIAR applications have been focused on postal and office automation addressing the needs for recognizing the content of scanned documents that were massively digitized with the ambition to work on paperless offices [5]. In the late 1990’s another important area of research in DIAR has been pushed by Digital Libraries initiatives whose digitized works needed to be indexed and understood [6]. Even if it is not easy to make a clear distinction of “historical documents” with respect to other documents, it is clear that works preserved in libraries printed before 1900 can surely belong to this category, that also includes other types of artifacts as we will discuss in Section 2.

The third wave of interest in the usage of ANNs in several application domains began a decade ago and is still going on. The change of paradigm pushed by Deep Learning (DL) architectures involved many disciplines and there are very few research areas that are not significantly impacted by the new paradigms. The DIAR research is not an exception. To demonstrate the impact of ANNs in general, and DL in particular, in the DIAR area we made an analysis of papers published in the proceedings of the ICDAR (International Conference of Document Analysis and Recognition) conference series that is organized biennially since 1991. In particular, inspecting the titles, we considered all the papers that used connectionist-based techniques. In Table 1 we summarize the outcome of this analysis. For each edition of the conference, we report the number of papers using ANN-based techniques and their percentage with respect to the total number of papers in the proceedings. By looking at the evolution of the second value over the years we can notice a relative maximum in 1995 with 6.5% ANN-based papers, corresponding to the second wave of research of ANNs and a minimum, below 2%, between 2007 and 2011. Since 2013 the share of papers using ANNs (actually deep learning grounded approaches) exponentially increased reaching a maximum of 85.5% in 2019.

Another interesting feature of papers using ANNs in the ICDAR proceedings is related to the addressed topics. In the last row of Table 1 we report the percentage of works considered in the second row and dealing with text recognition. Keeping in mind that computing these percentages on small numbers has very low statistical significance, it is clear that in the last years we can notice a significant reduction of the percentage of papers that use ANNs for text recognition. What once was a major research topic is now marginal and this is even more interesting when we observe that several papers in this category in the last years are related to text processing not involving document images or addressing scene text recognition.

Given these premises, we believe that this is the right time to analyze the major trends in the use of Deep Learning in a specific sub-area of DIAR research: the analysis and recognition of historical documents. Despite the subject of this work we are not going to make a retrospective historical analysis of this research in the past 20 years, but we focus on the very last few years, mostly since 2017, because of the larger number of papers and also because we aim to review state of the art works to help to identify useful research directions in this area and in related ones.

The paper is organized as follows. In Section 2 we first provide a general definition of historical documents that are the main target of the works discussed in this paper. Section 3 describes the most relevant tasks addressed by deep learning methods when processing historical documents. Common deep learning architectures are briefly summarized and discussed in Section 4 by looking at the architectures from the point of view of the input-output relation expected from the neural module. Datasets and other tools useful for conducting research on historical document processing are analyzed in Section 5. Final remarks highlighting general trends and future research directions are discussed in Section 6.

## 2. Historical Documents

History is defined through documents and is distinguished from pre-history because of the existance of written documents that describe in a permanent form different aspects of earlier life. While this consideration makes it easy to place a starting point for the definition of historical documents, (“an old document”) it is more difficult—if at all possible—to set an upper time limit (“how old should be a document to be historical?”). From this point of view, a document is historical since it can be studied from historians to analyze a given period of time. To better characterize the focus of our study, we will follow a more pragmatic approach and consider historical documents as those that are made on supports or by techniques not used anymore or that are more difficult to analyze than contemporary documents.

To better define the conceptual boundary of historical documents considered in our analysis we provide, in this section, a summary of items that are often considered historical by the DIAR research community.

### 2.1. Earlier Documents

Most ancient documents are written on different supports than paper: palm leaves, stones, bamboo scrolls, papyri. Writers of palm-leaf manuscripts used a support that is made out of dried palm leaves, used as writing materials in the Indian subcontinent and in southeast Asia dating back to the 5th century BCE and possibly much earlier. One of the oldest surviving palm leaf manuscripts of a complete treatise is a Sanskrit Shaivism text from the 9th-century, discovered in Nepal, now preserved at the Cambridge University Library [7,8,9]. The earliest surviving examples of wood slips or bamboo scrolls date from the 5th century BCE during the Warring States period. By the 4th century AD bamboo had been largely abandoned as a medium for writing [10]. Papyrus was first manufactured in Egypt as far back as the fourth millennium BCE and used for documents in the Diary of Merer, dated from 2560–2550 BCE [11].

### 2.2. Manuscripts and Incunabula

Manuscripts are a particular type of document, usually coming from Europe or “the western world” from classical period through the early centuries of the Christian era. Some of these manuscripts were also written without spaces between words (scriptio continua) or with really hard to understand handwritings often containing decorations or trimmings which make them especially hard for the untrained to read. Extant copies of these early manuscripts written in Greek or Latin and usually dating from the 4th century to the 8th century AD, are classified according to their use of either all upper case or all lower case letters. Several researchers addressed the analysis and recognition of these documents; even considering only those published in the ICDAR 2019 proceedings we can count nine papers ([12,13,14,15,16,17,18,19,20]).

After Gutenberg introduction in Europe of movable printing, early printed books became more and more popular [21]. These works are often referred to as Incunabula that is the plural of the Latin word incunabulum, a cradle. Evolving from its original meaning, incunabulum came to mean “place of birth” or “beginning”. In the world of books, the word incunabula refers to books that were printed using movable metal type up to the year 1500 [22].

### 2.3. Other Documents

In addition to documents whose main content is textual (described so far) other important items contain graphical information that needs to be addressed with different techniques and where the main aim is not to “read” and understand the text.

Some of these documents are preserved in archives that often contain handwritten documents. Generally speaking, archival documents are more diverse than documents in libraries and in most cases individual archived items are composed by few pages. Archival documents often describe financial information whose understanding requires different techniques than those used, for instance, to read a novel or a manuscript. Examples of archival documents written between 1470 and 1930 from nine different European archives are presented in Reference [23].

Historical documents coming from census, birth records, family records [24], and historical register books [25] contain structured information whose extraction allows scholars to reconstruct genealogies and perform demographic studies [26]. Several researchers addressed the recognition of these types of documents. Text detection and recognition in 31 historical U.S. maps (1866–1927) is discussed in Reference [27].

## 3. Addressed Problems

Different problems in the analysis of historical documents are addressed and solved in the research summarized in this paper. In this section, we highlight the main tasks considered in order to figure out what are the principal input-output relationships that need to be solved by the proposed deep learning techniques. The main tasks are organized according to a traditional pipeline in DIAR [1] where document images are first gathered (Section 3.1) then three main processing steps are performed: pre-processing to improve the quality of the images (Section 3.2), the identification of regions with homogeneous content with layout analysis techniques (Section 3.3), and finally the textual content recognition (Section 3.4). Sub-tasks are subsequently ordered on a top-down fashion in each of the following sections.

### 3.1. Building Collections in Digital Libraries

Historical documents, once digitized, are usually stored in Digital Libraries and archives. In order to index in the proper way documents in the collection, curators need to know some information about the archived items, like the writer of a manuscript and the date (or period) of production of manuscripts. To estimate the cost of transcription it is also useful to find out the number of records in archival documents. Deep learning techniques addressing these topics are discussed in the following.

#### 3.1.1. Writer Identification

Writer identification is the problem of coupling rows and pages from handwritten historical document images with their author and writer. This task has been addressed by Cilia et al. [28] in a work presenting an end to end system to identify writers in medieval manuscripts. The proposed system consists in a three-step model for detection and classification of rows in the manuscript and page writer identification. The row detection and classification steps are based on the combination of MobileNetV2 [29] fine-tuning and a custom CNN. The third stage is made of a weighted majority vote row-decision combiner which aims to link pages and authors. Siamese networks can also be used for writer identification and recognition as discussed in Reference [30].

#### 3.1.2. Manuscript Dating

Manuscripts are widespread in libraries and archives. Differently from more modern works, like printed books, the date of production of manuscripts is often unknown, however an automatic dating of these works is useful for an accurate archival in digital libraries.

The dating of historical manuscripts arises from the problem that is repeatedly faced when dealing with any manuscript with unknown origin and period of production. The main idea is to couple author’s writing skills, style and technique to a certain historical period. This is done either through the analysis of various features within the document pages or authors’ handwritings. Hamid et al. [15] present a deep learning based approach to effectively characterize the year of production of sample documents from the Medieval Paleographical Scale (MPS) dataset—a set of documents from the late medieval period (1300–1550). The proposed method relies on a convolutional-based model extracting features from handwriting document images patches. According to the authors this approach outperforms the traditional image processing-based feature extraction techniques.

More specifically, the results discussed by the authors are computed on the MPS dataset and the system performances are compared with other methods, mainly not deep learning based, which were previously used to solve the task. These traditional methods are mostly based on techniques designed to identify the texture of images like Fraglet and Hinge features [31,32], Quill features [33], clustering methods [34] and textural features [35]. The system proposed in Reference [15] greatly improves the results with respect to such previous works by at least a factor 2 (up to a factor 10), bringing the Mean Average Error (MAE) values to very low levels (from values ranging from 35.4% to 7.8% up to 3%), increasing the accuracy for the proposed task when evaluated on the dataset taken into account.

Deep learning methods for manuscript dating are also discussed in Reference [36], which highlights the importance and effectiveness of using pre-trained networks as features extractors from historical documents and then applying them by fine-tuning for different tasks.

#### 3.1.3. Estimation of Transcription Costs

Page-level analysis aims at providing some general information about an input digitized page. The simplest outcome that can be considered is page classification that is aimed at indexing pages for inclusion in digital libraries [37]. Page classification has been addressed with traditional ANNs (e.g., Reference [38]) and more recently with convolutional neural networks [39].

Several documents related to census and register books contain handwritten records describing facts about families. When dealing with large collections of these documents, an accurate count of the number of records in the collection can provide valuable information to assess the amount of data available within document images [26]. To quantify the number of records (and therefore estimate the transcription costs), record counting at the page level can be considered. Capobianco et al. [26] addressed this problem by using a deep architecture fed with a page image and providing as output a numerical value corresponding to the number of records in the page. The contribution is twofold: first, authors propose a tool for semi-automatically generating synthetic handwritten documents containing generally structured records; second, they demonstrate that training a deep architecture with artificially generated pages can be a very good and efficient way to estimate the number of records in real scanned historical documents.

Given the increasing trend towards transforming digital libraries into places where users can quickly find information and books in an efficient and effective way, it is essential to estimate the time required for transcribing the textual content. In Reference [40] the authors propose a segmentation-based model for the estimation of the time needed to transcribe a large collection of historical handwritten documents when the transcription is assisted by a keyword spotting system following the query-by-string approach. The model has been validated by comparing its estimates with the actual time required for the manual transcription of pages from the Bentham dataset [41].

### 3.2. Pre-Processing

After digitization, the first step in a traditional DIAR pipeline is the input images pre-processing. This task is aimed at improving the document quality either for a better human inspection of the work or for improving the automatic recognition of subsequent processing steps. Because of the nature of historical documents this step is particularly relevant in this area as demonstrated by the diverse papers related to pre-processing of historical documents. Two main related operations are performed for preprocessing: document image enhancement (including image denoising and image restoration) and document binarization.

#### 3.2.1. Image Enhancement

Historical document denoising is one of the most challenging steps in the field of image processing and computer vision. Neji et al. [22] propose a novel end to end adversarial autoencoder (AAE) which generates clean images and shows adversarial autoencoder’s power when used in historical document denoising. The proposed AAE uses a generative adversarial networks (GAN) so as to suit the aggregated posterior of the hidden code vector of the autoencoder with an arbitrary prior.

In the case of historical document restoration the main purpose is to improve the image quality to ease the subsequent document analysis steps. Despite of recent breakthroughs in the accuracy of isolated character recognition using deep neural networks, OCR systems almost fail to recognize character patterns when they are severely degraded, especially those in historical documents. Moreover, the lack of sufficient training patterns, due to the annotation cost, increases these difficulties. Nguyen et al. [42] propose a character attention generative adversarial network named CAGAN for restoring heavily degraded character patterns in historical documents so that OCRs can improve their accuracy and help archeologists to decode them. The network is based on an U-Net like architecture [43], and it is trained by a loss function which includes the common adversarial loss as a global loss and a hierarchical character attentive loss as local loss term. A different pipeline for the same task is presented by Uzan et al. in Reference [44]. This work aims to restore fragmentary letters in ancient manuscripts, a fundamental step in order to read them. The authors present a method to complete broken letters in the Dead Sea scrolls, which is based on PixelCNN++ [45], an autoregressive generative model designed for image restoration.

#### 3.2.2. Image Binarization

Another important task in document image pre-processing is binarization. This consists in the transformation of an input color or gray level image into a black and white one. This task is often performed to minimize the impact of physical document degradations as non-uniform background, stains, faded ink, ink bleeding through the page, and uneven illumination on document images. The binarization process separates the document content from these noise factors by classifying each pixel as either foreground or background. This is a well-studied problem, even for highly degraded documents, as evidenced by the popularity of the Document Image Binarization Content (DIBCO) competitions [46].

Tensemwyer et al. [46] formulate binarization as a pixel classification task and to do so apply a Fully Convolutional Network (FCN) architecture capable to operate at multiple image scales, including full resolution, for better generalization. In particular, the suitability of the proposed deep learning based approaches is confirmed and reinforced by the fact that the experimental results are better, in terms F-measure, Distance Reciprocal Distortion (DRD) and Peak Signal to Noise Ratio (PSNR), than those achieved by at least 4 winners of the previous 7 editions of the DIBCO and H-DIBCO competitions (F-measure from 91.5% up to 94.9%, average 2% improvement for both PSNR and DRD). Moreover, Reference [46] represents one of the first works that succeed in improving the methods previously used, mainly based on page, border and line analysis, through deep learning techniques.

**Figure 1:**
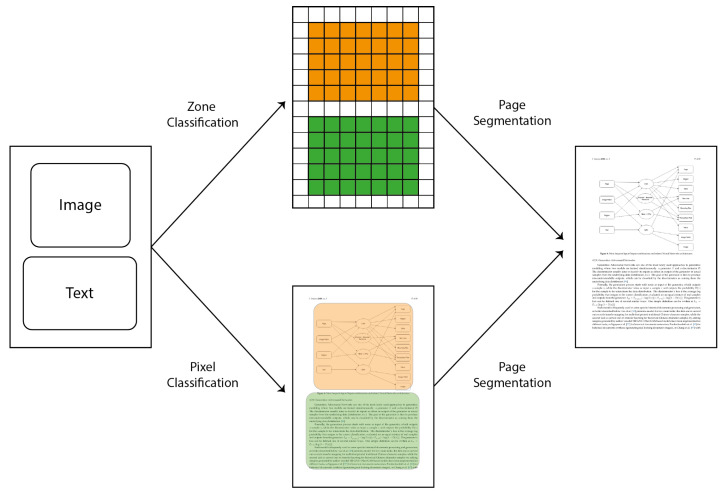
Zone (or patch) classification compared to pixel classification.

From a broader perspective, pixel classification aims at providing a label to each pixel of an input image and can be contrasted with zone or patch classification where a single label is provided for each patch in the image (Figure 1) [47]. While previous solutions for pixel classification were based on a sliding window approach, where the same neural network is used to compute the output at different positions in the page [2], deep learning based solutions use single networks for performing the pixel labeling relying on the weight-sharing of convolutional layers with clear advantages in terms of processing speed during recognition.

### 3.3. Layout Analysis

Layout analysis is the process of identifying and recognizing the physical and logical organization of scanned documents. The typical processing chain in most DIAR applications starts with pre-processing operations that are aimed at cleaning noisy images and are then followed by layout analysis and by character/symbol recognition. Machine learning techniques have been widely used for layout analysis [47] and this is still one important testbed for applications of deep learning techniques in DIAR. The sub-tasks described in this section are organized in a top-down way. We first describe techniques where tables are identified in whole pages, then approaches aimed at locating whole text lines or their baselines.

#### 3.3.1. Table Detection/Recognition

In the last years online access to archival documents has been increased significantly. The availability of documents such as register or census books containing either text and tables, which can be either fully drawn and written by hand or printed, has stimulated the research on information extraction from tabular documents. Clinchant et al. [25] address in their work the problem of table understanding: recognizing the structural organization of tables to extract information of interest. In more details, the authors first explain and model the problem of row and column detection, then compare two machine learning approaches for the detection of these table elements. They evaluate the model on death records provided by the archives of the diocese of Passau, presenting good results. In the paper several deep and not deep machine learning methods are compared for the detection of table or register books elements at different levels of granularity (row-column, cell, sentence, word, letter). In particular the comparison takes into account Conditional Random Field (CRF) [48], Graph Convolutional Networks, logit, logit followed by a convolutional layer, deep CNNs. According to the experiments performed deep learning-based approaches have raised better results in terms of Precision, Recall, F1-score and BIESO (Beginning, Intermediate, End, Single word entity and Outside) tagging accuracy, namely getting from 89% to 95% up to 92% to 96% for all those measures [25]. Tables in census records have been also analyzed in Reference [26].

In the last few years an important research topic in DIAR has been the analysis of tables in contemporary documents by using graph neural networks [49] or dealing directly with PDF documents [50]. It is reasonable to expect that these techniques could be used also to deal with historical documents.

#### 3.3.2. Text Line Segmentation

**Figure 2:**
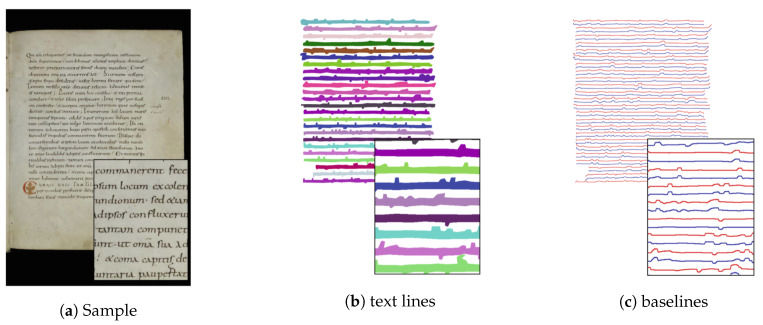
Different approaches for the identification of text lines in the manuscript in (**a**): text line segmentation (**b**) and baseline detection (**c**) [51].

Text lines segmentation is one central task for the analysis of manuscripts and other historical documents. While the segmentation of text lines in printed documents is relatively easy, the segmentation of historical handwritten text is particularly difficult because of the irregular text line orientation and of the presence of various artefacts (like capital illuminated letters and marginalia, that is, notes added to the page). Another aspect of manuscripts is that these were written trying to use as few pages as possible; therefore the text lines are closer one to the other with respect to modern documents. Text lines segmentation aims at identifying the area corresponding to each text line (Figure 2b) and has been addressed with several deep learning based approaches.

Chen et al. [52] propose a simple CNN having only one convolution layer. The text lines segmentation is achieved by means of super-pixel labeling where each pixel is classified as background, main text, decoration, or comment. The input to the classification is a patch centered on the super-pixel. Experiments have been performed with several well-known datasets, such as the George Washington, St.Gall, and Parzival (see Section 5.1). In all the results discussed high accuracy is reported. For a better understanding, the proposed model is compared with CRF and Multilayer Perceptron (MLP) as pixel classifiers, obtaining accurate results. In more details, superpixel labeling mean accuracy and Intersection over Union (IU) are improved by the proposed method by 1% up to 4%, up to more than 90%,depending on the tested dataset [52].

Pastor-Pellicer et al. [53], instead, address the text lines segmentation by estimating the Main Body Area (MBA) for each text line. The MBA is the area between the baseline and the corpus line. Two classification levels are considered. In the first level, each pixel of the image is classified (by considering a sliding window centered on it) into three classes—background, text block or decoration. By using this pixel labeling text regions are identified. A second CNN classifies pixels in text blocks by observing a sliding window centered on the pixel and labeling it as belonging to the MBA or not. The proposed approach is mainly evaluated and tested with the Parzival and St. Gall datasets. When analysing the compared results, the proposed one is the only based on deep learning and it shows an improvement (more or less consistent) with respect to any of the works with which it is compared. Alternative approaches are based on non deep learning-based solutions ranging from historical page analysis performed by anisotropic scale-space smoothing, going through methods based on the identification of connected components, up to clustering methods for text lines segmentation. The comparison is also made with a text-lines extraction technique based on Dynamic Multilayer Perceptron (DMLP) classifiers, which obtains similar results.

A variant of deep Fully Convolutional Networks (FCNs) with dilated convolutions is adopted in Reference [54]. In this work the input and output sizes match and the network performs a pixel labeling even without an explicit sliding window. The FCN is trained to identify the X-height (distance between the baseline and the mean line) of text lines, with an output that is similar to the MBA labeling proposed in Reference [53]. In this case the results are compared with methods based on machine learning. Generally speaking, the method comes very close to state of the art results, improving some of them in terms of Precision, Recall and F1 score (performances from 88% to around 95%) and getting close to others based on convolutional architectures such as U-net or its variants.

Alaasam et al. [12] perform a patch classification by using a Siamese network that compares a pair of patches and gives as output their similarity. In this way it is possible to cluster the patches in each page in three classes: main text, side text (annotations), and background. The approach is tested on 8 pages (containing around 4000 patches) taken from different Arabic historical manuscripts. Results have been evaluated in comparison with Reference [55] and Reference [56]. In particular, Bukhari et al. [55] uses an MLP-based approach, while Reference [56] presents an FCN for curved lines layout analysis. Working on patch level instead of page level, the proposed method outperform these works in both the main text and side text segmentation, improving accuracy for particular pages layout analysis too, bringing F-measure for main text and side text analysis respectively from 95% to 98.5% and from 94% to 96.8% [12].

Different text lines are located in Indic historical documents in Reference [14] by using a deep model based on a Mask R-CNN with a ResNet-50 backbone. The different task of instance segmentation (that separates individual objects in the page, e.g., each text line) with respect to semantic segmentation (that aims at identifying pixels belonging to a given object type, e.g., text lines) is taken into account and discussed in the paper. With respect to other approaches more classes are considered among the object types: Character line segment, Page boundary, Hole, Boundary line, Character component, and Physical degradation.

Text segmentation in non-alphabetic writings requires the identification of individual symbols rather than text lines. Watanabe et al. [20] propose a character segmentation method composed by the application of a FCN and a post-processing phase applied to its outputs. There are three main features of the proposed method: it can directly process input images including multiple text lines making a text lines segmentation process unnecessary, it is character recognition-independent, and it is robust to variations in character size, characters gaps and overlappings. An accuracy of 95% has been reached for the character segmentation task, tested on real images of Japanese historical handwritten official documents.

#### 3.3.3. Baseline Detection

Baseline detection is a slightly different approach with respect to text line segmentation previously discussed. In the latter case the task is to identify the individual text lines (Figure 2b) while in the former one the aim is to identify the line below each text line (Figure 2c). The main reason for considering two different tasks is the annotation cost for labeling data used for training. Drawing a line below a text line is clearly easier (and quicker) than enclosing all the pixels belonging to the line.

A convolutional neural network based on U-Net is used in Reference [57] for baseline detection and text lines recognition within historical document images. More precisely, the proposed BL-net model is a residual U-net, tested on different configurations and number of hidden layers. It has been evaluated predicting baselines on images at different scales and with different data sets, and performs equally or better than the compared works, achieving results of accuracy close to 99% (1% improvement with respect to the HistDoc best [57]). A similar approach is considered by Reference [58] that still uses an architecture based on U-Net for baseline identification in complex layouts as well as curved and arbitrarily oriented text lines. In this case, tests have been performed in the CBAD 2017 (ICDAR 2017 competition for baseline detection) dataset and therefore results are compared, in terms of precision and recall, with the participants of such competition. The proposed approach improves precision, recall and F-value of all these works. Another U-Net style architecture implemented for the identification of the baseline in manuscripts has been proposed also in Reference [59].

### 3.4. Character and Symbol Recognition

Character recognition is the problem of understanding and recognizing language characters from different idioms, either handwritten or printed. In the earlier years of applications of Artificial Neural Networks to perform character recognition most methods addressed western languages (often English) [2]. This was clearly due to large datasets available for research. In the latest years several digitization programs in many countries around the world made the study and application of different character recognition techniques on various languages and scripts possible.

#### 3.4.1. Keyword Spotting

One task closely related to text recognition is *keyword spotting* where occurrences of a query word are located in digital documents. The use of keyword spotting is appropriate in the field of historical documents since recognition systems can fail because of low quality of handwriting and lack of availability of suitable dictionaries used to check the results of handwriting recognition. One application of CNN architectures for keyword spotting in handwritten documents is proposed in Reference [60] and is tested on different historical datasets, including the George Washington [61] and the Esposalles [62]. Keyword spotting on handwritten historical documents is addressed also in Reference [63].

**Figure 3:**
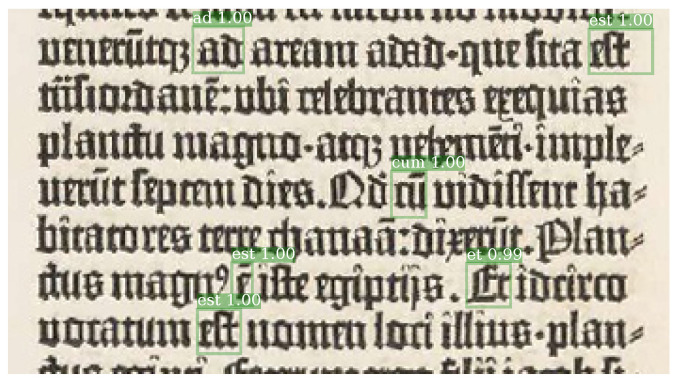
Keyword spotting in a fragment of Gutenberg’s Bible [21].

Convolutional architectures initially proposed for object detection in natural images (Faster R-CNN) are trained in Reference [21] to locate words in pages of the Gutenberg’s Bible (Figure 3). The authors train one initial model to recognize generic words and hyphens in pages. Using the model prediction a second model able to detect some landmark words is trained. The landmark words are frequent words that can be found with good accuracy and that are used to align the image with an inaccurate page transcription.

#### 3.4.2. Text Detection

Historical maps often contain both textual and graphical information which represent different geographic or political features and spatial scales. Such complex historical documents involve several unique challenges: text can appear in nearly any orientation, many sizes, and widely spaced with graphical elements or even other text within the local field of view. In this context it is important to perform text detection and recognition without assuming that the text is regularly organized in text lines. Many previous works present or use hand-crafted algorithms for dealing with such complexities, while Reference [27] and Reference [64] bring map document processing into the deep learning era, avoiding the need for complicated algorithms design. It has been done at first in Reference [64] by a unified probabilistic model, containing a CNN word recognition module, to leverage the mutual information between text labels and styles and their geographical locations and categories, and then in Reference [27] by designing a Convolutional Recurrent Neural Network (CRNN) which behaves at first as a features extractor performing ResNet50 fine-tuning and then as a text recognizer based on bidirectional LSTM. The authors demonstrate that with sufficient training data, deep learning systems can be efficiently used to extract text from complex historical maps images.

Locating and recognizing textual information in natural images is the main objective in scene text detection, one research topic that received a significant attention in the last few years in the DIAR field (e.g., References [65,66]). Using these techniques for recognizing text in graphical historical documents is a relevant research direction to be taken into account.

#### 3.4.3. Character Recognition

Cai et al. [67] deal with historical Chinese character recognition, facing problems including low image quality and lack of labeled training samples. The authors propose a GAN-based transfer learning method to ease the solution of these problems. The discriminator is based on a CNN, while the structure of the generator is a CNN based encoder-decoder. Experiments are conducted on two tasks to evaluate the performance of the proposed method (TH-GAN): the first task is carried out on style transfer mapping for multi-font printed traditional Chinese character samples, while the second task is carried out on transfer learning for historical Chinese character samples by adding samples generated by TH-GAN. Experimental results show that the proposed TH-GAN is effective.

Clanuwat et al. instead, deal with Kuzushiji handwritings recognition [13]. Kuzushiji, a cursive writing style, had been used in Japan for over a thousand years starting from the 8th century. However, following a change to the Japanese writing system in 1900, Kuzushiji has not been included in regular school curricula and so not inherited by nowaday’s generations. To tackle these challenges, the authors propose KuroNet, an end to end model which recognizes an entire page of text by using a residual U-Net architecture which predicts the location and identity of all characters given a page of text. Such system is able to successfully recognize a large fraction of pre-modern Japanese document. Ly et al. [68] present an end to end recognition system for Japanese historical documents. The system has two main modules: a dense CNN for extracting features, and a Long Short Term Memory (LSTM) decoder with attention model for generating target text. Nagai et al. [69] present a novel method for single Japanese historical cursive text lines recognition. The method reaches more than 95% accuracy for texts consisting of 46 Hiragana characters and 84.08% accuracy when including thousands of Kanji characters. Both of them are state-of-the-art accuracy.

Besides previous applications, the research on the recognition of handwritten text in western languages is not yet completely solved. One of the most significant research topics in this domain is the development of techniques to train an handwriting text recognition (HTR) system with few labeled data. Chammas et al. [70] demonstrate how to train an HTR system with few labeled data. Specifically, they train a deep convolutional recurrent neural network on only 10% of manually labeled text line data from a dataset and perform an incremental training procedure that covers the remaining 90%. Performances are increased by a multi-scale data augmentation process.

## 4. Neural Architectures and Their Applications

Since the proposal of the first deep learning architectures based on convolutional neural networks [4], several techniques have been proposed over the years. In this section we do not aim at describing in detail all these different architectures since several books and research papers have been recently published (e.g., Reference [71]). Rather, we want to focus on approaches that have been demonstrated to be suitable for addressing historical document recognition. To this purpose, we first discuss in Section 4.1 the different input-output relations useful in historical document recognition according to the analysis performed in Section 3. We subsequently describe the different architectures that can be used to train the above input-output relations in Section 4.2. In Section 4.3 we the summarize the types of architectures that have been used to obtain the desired input-output relations.

### 4.1. Input-Output Relations

According to Reference [2] the different uses of artificial neural networks for processing scanned documents can be described considering the different input and output information that are required in the specific application domain. Updating this analysis to current deep learning approaches for recognizing historical documents, we can summarize the main trends as discussed in the following and summarized in Table 2. Four main representations of the input document image can be considered: the whole page, fixed-size patches moved across the page, regions with homogeneous content, and individual text lines.

Various types of information can be computed from complete pages. Pre-processing (binarization, image restoration) is one typical task where the input and output have the same size and correspond to the whole page. In this case the desired operation corresponds to an image filtering and can be used for document image binarization [46], image generation [72], and pixel labeling [74] among other tasks. Most techniques in layout analysis identify regions with a uniform content, like paragraphs. As an example, in Reference [56] and Reference [75] the regions extracted correspond to the main text or to annotations in Arabic manuscripts. In Reference [76] text, annotations, titles, and illustrations are identified in historical documents. One related task is table detection in register books [25]. One widely addressed segmentation target is related to the identification of individual text lines that can be achieved both by segmenting the text lines (Section 3.3.2) or by identifying their baseline (Section 3.3.3). In some contexts the textual content in the document is more sparse like in historical maps [27] or in keyword spotting [40]. In these cases the output information is based on suitable bounding boxes around the items of interest. When the overall aim is to extract the textual information from historical documents, then we need text transcription that is addressed in several related works (e.g., References [13,18,60,63,70,79,80]). Global information (numerical values) corresponding to each page can be computed as well for instance to date the document [28] or to identify the number of records in register books [26].

Some methods take as input an image patch that is translated over the input image and that can produce as output a modified patch for image denoising [22], document restoration [42] or letter restoration [44]. The output information computed from each patch can be also used for document dating [15] or writer identification [28]. It is important to notice here that several CNN-based approaches follow implicitly a similar paradigm since in the first layers the convolutional filters are moved across the image to extract the same features in different positions (Section 4.2.1).

Text recognition can be also addressed by looking for the content in a region with various approaches that are often similar to those proposed for text recognition in whole pages ([68,81,82,83]). The last combination of input-output relations that we consider is textual information that is transformed into a simulated historical document for performing data augmentation for training deep learning algorithms [84].

### 4.2. Deep Learning Architectures

In the last few years, several deep learning architectures have been proposed to address different application areas. Since most problems in historical document analysis concern the recognition of scanned documents it is reasonable that a large fraction of works rely on various flavours of convolutional neural networks (Section 4.2.1, Section 4.2.2, Section 4.2.3, Section 4.2.4, Section 4.2.5 and Section 4.2.6). To address image-to-image mapping (or more generally filtering) also autoencoder-based approaches have been used (Section 4.2.5). When taking into account the textual content of documents, that can be considered as a sequential flow of information, recurrent neural networks are natural architectures to be considered in particular in the latest LSTM approaches (Section 4.2.7). The last family of architectures used for historical documents are generative adversarial networks that have been used for training set generation (Section 4.2.8).

#### 4.2.1. Convolutional Neural Networks

Convolutional neural networks (CNNs) are a specialized kind of neural network designed for processing data that has a known, grid-like topology [71]. The basic idea of CNNs is to simulate the natural functioning of the animal visual cortex. For this reason, that is to say to exploit the existing spatial relation between the data, the convolutional networks are not completely connected (the units of a layer are not connected to all the units of the next layer). Examples include time-series data, which can be thought of as a 1D grid taking samples at regular time intervals, and image data, which can be thought of as a 2D grid of pixels. Convolutional networks have been tremendously successful in several applications in particular in the area of computer vision. The name comes from the use of a mathematical operation called convolution that is a specialized kind of linear operation. Convolutional networks are simply neural networks that use convolution in place of general matrix multiplication in at least one of their layers.

CNNs have been widely used for historical document image processing and recognition and basic convolutional layers form the backbone of several more complex architectures that are summarized in the following. CNNs are very effective in reducing the number of parameters without losing on the representation power of learned models and this is why images complexity can be reduced in informative, but smaller, feature maps extracted by convolutional layers.

Learning useful feature extraction in the first layers of CNNs requires a significantly large training set. Because datasets available in the area of historical documents (Section 5.1) are often not large enough, transfer learning strategies are required to successfully tackle the various problems described in this paper. The key idea is to transfer and generalize the learning of one task to another similar one. The concept of transfer learning is specially useful when the amount of available data regarding a specific problem is not large enough to train a deep CNN from scratch. In such scenarios, a pre-trained network as ImageNet can be employed either as features extractor from the images under study, or by adapting its last layers to fine-tune the network itself. Hamid et al. [15] compare different CNN pre-trainings for manuscripts dating, Weinman et al. [27] use ResNet50 as a convolutional backbone for text detection and recognition in historical maps. The analysis of the effect of network pretraining on ImageNet for varous tasks in historical document analysis is discussed in Reference [36]. The set of tackled tasks include character recognition, style classification, manuscript dating, semantic segmentation, and content-based retrieval.

#### 4.2.2. Siamese Neural Networks

**Figure 4:**
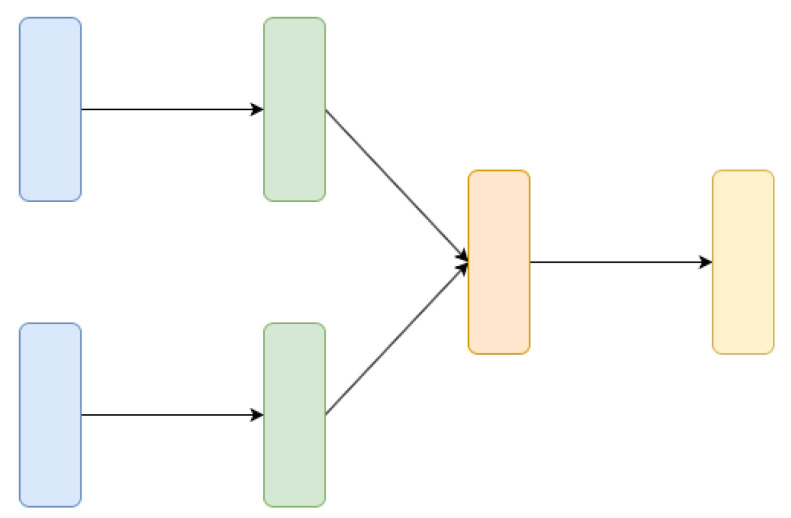
Simple Siamese Neural Network architecture. Layers with the same color share the weights.

Siamese Neural Networks are neural networks containing two or more identical subnetwork components [85]. Those components share the same weights while working in tandem on two different inputs to compute comparable outputs, and they are typically used for comparing similar instances in different type sets. A Siamese neural network may look as like as in Figure 4. It is important to underline that not only the architectures of the subnetworks are identical, but also the weights are shared and learned together and this is why the the network is called siamese. The main idea behind siamese networks is that they can learn useful data descriptors that can be further used to compare the inputs of the respective subnetworks. Siamese networks are used in Reference [12] to extract text lines by comparing document images patches and estimating their similarity.

#### 4.2.3. Fully Convolutional Networks

The basic concept behind Fully Convolutional Networks (FCN) is to contain only convolutional layers [86]. FCNs do not have any of the fully connected layers at the end of the architecture, which are typically used for classification. Instead, these networks use convolutional layers to perform pixel classification, computing an output having the same width and height as the input image, but with a number of channels equal to the number of classes. Convolutional neural networks use pixel stride of receptive fields to reduce the size of feature maps and extract features that are expected to represent the input from an high level point of view. Because of this property of CNNs some strategies need to be considered for obtaining an output prediction of the same size of the input. One possibility is the use of upsampling that can be obtained with bilinear interpolation or that can be learned with backwards convolution also called deconvolutions [86]. Since the oputput predictions have the same size of the input image, FCNs are very efficient and effective—and therefore widely used—for tasks such as semantic segmentation and pixel classification. Models that comply with the above mentioned features have also been used in the field of historical documents processing and understanding, as summarized in the following. Prusty et al. [14] use FCN for instance segmentation of text lines and other areas in historical documents and a similar approach is used in Reference [76]. Individual Japanese characters are segmented with FCN in Reference [20]. Another task addressed with FCN is image binarization, that clearly requires an output representation matching the input size [46,56].

#### 4.2.4. U-Nets

**Figure 5:**
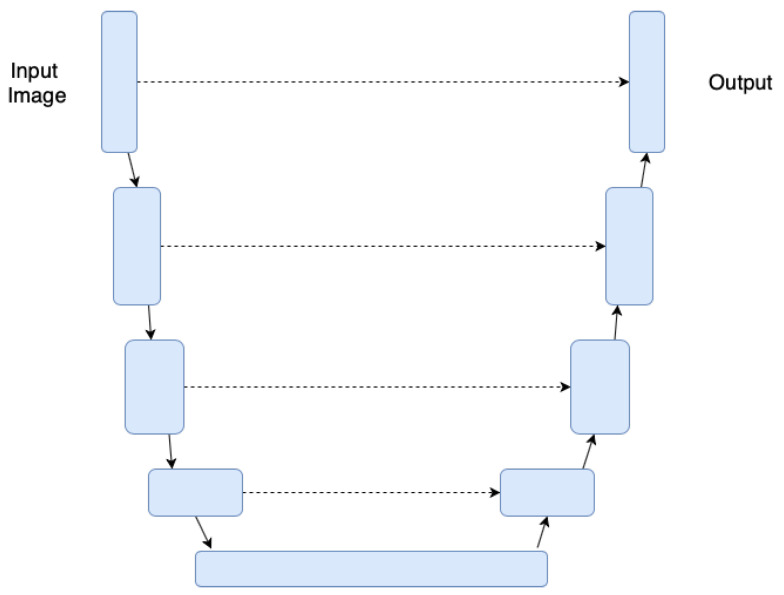
General schema of Unet architecture: input and output have the same size.

U-Net is a type of FCN very suitable for image segmentation and initially proposed for biomedical image segmentation [43]. Likewise other FCNs its goal is to predict each image’s pixel class. The network architecture is sketched in Figure 5. It consists of a contracting path (left side) and an expansive path (right side). The contracting path is made of a standard convolutional network. It consists of the repeated application of two 3×3 unpadded convolutions, each activated by ReLU and a 2×2 max pooling downsampling operation—each downsampling step doubles the number of feature channels. On the other hand, every step in the expansive path consists of an upsampling of the feature map followed by a 2×2 convolution that halves the number of feature channels, a concatenation with the correspondingly cropped feature map from the contracting path, and two 3×3 convolutions, each followed by a ReLU. The final 1×1 convolutional layer is used to map each feature vector to the desired number of classes—in total the network has 23 convolutional layers [43].

This architecture was designed and created to solve the task of image segmentation within the medical field; examples include the segmentation of brain images, internal organs, and various types of cells. Later on, given the effectiveness of this method when used for the segmentation task, the architecture has been used in many other areas. One of these is most certainly the analysis of historical document images and in particular the document segmentation.

In many cases, several authors have been inspired by the U-net architecture—often resized or adapted to the amount of available data and the specific nature of the images involved—to implement models which are derived from it, and then applied to solve the most diverse tasks. U-Net is used for some tasks in a fashion similar to FCN. Text line segmentation is addressed by Reference [77] while the related task of baseline detection is discussed in Reference [57]. The detection and recognition of Kuzushiji Japanese characters is proposed in Reference [13].

#### 4.2.5. Encoder-Decoder Networks

Object detection in images gained a significant interest in the latest years because of the introduction of powerful deep learning based techniques. Traditional methods were based on handcrafted algorithms for region proposal and features extraction from the image followed by trainable classifiers. Modern architectures on the other hand address the region selection, feature extraction, and classification within a single trainable architecture outperforming previous approaches. Several convolutional architectures have been recently proposed for addressing object detection: Faster R-CNN, R-FCN, Multibox, SSD, and Mask-RCNN, just to mention a few [87].

Applications of object detection models for historical documents range from keyword spotting in early printed works [21] by using Faster R-CNN, to the location of different text lines in Indic historical documents considering a Mask R-CNN architecture [14].

#### 4.2.6. Deep Models for Object Detection

**Figure 6:**
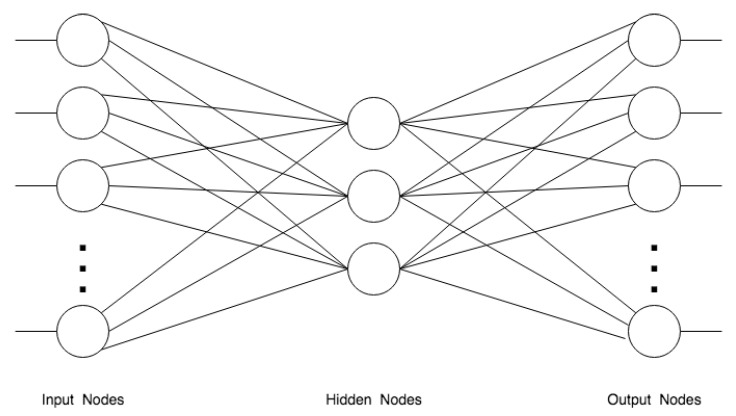
Autoencoder architecture: input and output have the same size.

Autoencoders and Encoder-Decoder Networks are artificial neural networks that learn how to efficiently compress and encode data and how to reconstruct it back from the reduced encoded representation to a representation that is as close as possible to the original input. These neural networks, by design, reduce data dimensions by learning how to ignore the noise in data. Autoencoders were first introduced in the 1980s by Hinton and the PDP group [3] for unsupervised training of backpropagation networks, by using the input data as the teacher [88]. The simplest form of an autoencoder is a feedforward, non-recurrent neural network similar to individual layers in multilayer perceptrons—having an input layer, an output layer and one or more hidden layers connecting them—where the output layer has the same number of neurons as the input layer, and with the purpose of reconstructing its inputs, minimizing the difference between input and output, as shown in Figure 6.

As just mentioned, autoencoders are unsupervised learning models which leverage neural networks for the task of representation learning. Specifically, they are designed as a neural network architecture imposed to have a bottleneck in it, which forces a compressed knowledge representation of the original input. Convolutional Autoencoders similarly to FCN and U-Net have the same dimensions of input and output. Convolutional autoencoders to learn features from historical document images and classify them are used in Reference [89]. CNN for feature extraction and LSTM-based encoder and decoder for text lines recognition is discussed in Reference [82]. A convolutional autoencoder to learn features in document images used by an Support Vector Machines (SVM) to perform page segmentation is proposed in Reference [90]. Another typical use of autoencoders is image denoising—in this context Incunabula are processed by Reference [22].

#### 4.2.7. Recurrent Neural Networks

Recurrent neural networks (RNNs) are a type of neural network model containing a self-connected hidden layer. This kind of model is able to process a sequence of arbitrary length by recursively applying a transition function to its internal hidden state vector of the input sequence. The activation of the hidden state for each time step is computed as a function of the current input and the previous hidden state [91]: one benefit of this recurrent connection is that a “memory” of previous inputs remains in the network’s internal state, allowing it to make use of past context [92]. Nevertheless, the range of contextual information that traditional RNNs can access is limited, and this is due to the fact that the influence of each input on the hidden layer, and therefore on the network output, either decays or blows up exponentially as it cycles around the network’s recurrent connections. This problem has been faced through LSTM, a particular RNN model designed to specifically address this issue of learning long-term dependencies where the LSTM contains an inner separate memory cell that updates and exposes its content only when deemed necessary [91].

LSTM is widely used for many tasks and one of them is definitely historical document processing and understanding. It has been used for classification tasks as in Martinek et al. [93], which presents a CNN-LSTM model for manuscript character classification, or as in Chammas et al. [70] for baseline identification. In Reference [70] the authors present a deep Convolutional Recurrent Neural Network (CRNN) inspired from the VGG16 architecture used for image recognition. The architecture includes a stack of convolutional layers followed by bidirectional LSTM ones with 256 units per layer, spatial pooling and ReLU activation after each convolution. Bluche et al. [83], instead, perform both line segmentation by computing attention weights on the image representation and line transcription. Ly et al. [81] implement an end to end model of deep CRNN for handwritten Japanese text lines recognition. The model is made of three parts—a convolutional feature extractor from a text line image, recurrent layers employing a bidirectional LSTM to predict the feature sequence frames and a transcription layer using Connectionist Temporal Classification (CTC) to convert these predictions into the label sequence. Messina et al. [94] implement the usage of Multidimensional Long-Short Term Memory Recurrent Neural Networks (MDLSTM-RNN) for non-segmented handwritten Chinese character recognition, while Moysset et al. [95] present an approach for full page text recognition. In this case, text lines are recognized in two steps: an FCN detects where to start to recognize a text line whitin the page and a multidimensional LSTM performs text recognition and decides when to stop the process.

#### 4.2.8. Generative Adversarial Networks

Generative Adversarial Networks are one of the most lately used approaches to generative modeling where two models are trained simultaneously: a generator *G* and a discriminator *D*. The discriminator usually aims to classify its inputs as either an output of the generator or actual samples from the underlying data distribution p(x). The goal of the generator is then to produce mis-understandable outputs, which can be classified by the discriminator as coming from the underlying data distribution [96].

Formally, the generation process starts with noise as input of the generator, which outputs a sample *x*, while the discriminator takes as input a sample *x* and outputs the probability D(x) for the sample to be taken from the data distribution. The discriminator’s loss is the average log probability that assigns to the correct classification, evaluated on an equal mixture of real samples and outputs from the generator: $L_{D}=E_{x∼p(x)}\left[-\log D(x)\right]+E_{x∼G}\left[-\log\left(1-D(x)\right)\right]$. The generator’s loss can be defined in several ways. One simple definition can be written as $L_{G}=E_{x∼G}\left[\log\left(1-D(x)\right)\right]$.

Such model is frequently used in some specific historical documents processing and generation, as better described below. Cai et al. [67] present a model for two main tasks: the first one is carried out on style transfer mapping for multi-font printed traditional Chinese character samples, while the second task is carried out on transfer learning for historical Chinese character samples by adding samples generated by author’s model TH-GAN. Other GAN-based models have been implemented for different tasks, as Nguyen et al. [42] for historical documents restoration, Pondenkandath et al. [84] for historical documents synthesis (generating real-looking document images), or Chang et al. [97] with an hierarchical GAN for Chinese handwritten characters imitation or Reference [98] for Chinese hanwritings identification using CycleGAN [99].

### 4.3. Input-Output Combinations and Related Neural Networks Architectures

**Figure 7:**
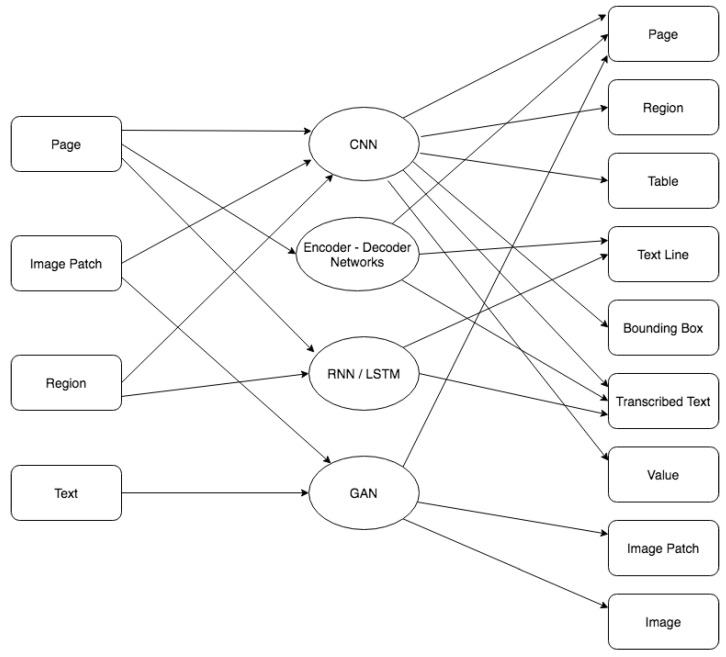
Most frequent Input-Output combinations and related Neural Networks architectures.

We analyzed in Section 4.1 the most common combinations of input and output information and in Section 4.2 the most used deep architectures used when processing historical documents. Figure 7 summarizes what types of architectures have been used to implement the expected input-output relation in the literature analyzed in this paper. This synthetic diagram can be used as a reference to point out what types of architectures can be considered to solve specific tasks.

## 5. Experimental Environment

Effective and efficient design of solutions based on deep learning techniques requires a solid experimental environment that includes two main components: labeled datasets and training platforms. Another component that fuels this research is the organization of specific competitions that are instrumental also for building valuable datasets. When the collection of large datasets is not possible, then transfer learning strategies and the generation of synthetic training data can be considered as well. The research for the historical document analysis and recognition is not an exception, as summarized in this section.

### 5.1. Datasets

In the last years several datasets have been proposed for supporting the research on historical document recognition as discussed in the following and summarized in Table 3. It is important to remark that all these datasets are not a mere collection of images and transcriptions, but are studied and annotated by scholars in the Humanities, expert in the respective area.

Archives are an important source of material for the research in historical document recognition. The George Washington dataset is a well known collection of scanned pages of handwritten letters from George Washington and his assistants. It consists of 20 pages of the collection annotated at the word-level and used in particular to test keyword spotting algorithms [61]. Marriage records and other demographic data are another source of data. One example is the Esposalles dataset [62] that is made by structured handwritten pages gathered from marriage license books spanning 500 years. The dataset is split into two parts—the Index that contains 29 pages of indexes of the books and the Licenses that contains 173 pages with actual records describing individual marriages. The pages are annotated at the word level. More recently, Grüning et al. [23] proposed the READ-BAD dataset used for baseline detection in archival documents. It contains 2036 archival document images from different locations and time periods with several page layouts and degradations.

Manuscripts, often held in libraries, are another relevant kind of documents. DIVA-HisDB [100] is a widely used accurately annotated large dataset of challenging medieval manuscripts written in Latin and early Italian. It has been used for the evaluation of several DIAR tasks. The dataset consists of 150 pages of three medieval manuscripts with difficult layouts. The groundtruth information provided for layour analysis tasks has been collected with the support of experts in medieval studies. The DIVA-HisDB dataset is used for training and evaluating several tasks, like layout analysis, text line segmentation, binarization and writer identification. The St. Gall [101] and Parzival [102] datasets have been also often used to design and test techniques for layout analysis and text line segmentation.

Non-latin scripts are becoming more and more relevant in the research on historical documents. VML-HD is a dataset of arabic documents [103]. It is based on five books written by various authors between 1088 and 1451. A total of 680 pages are fully annotated on the sub-word level. The Pinkas Dataset [104] is a dataset of medieval handwritten Hebrew manuscripts annotated at word, line and page level by an historical scholar. The research on convolutional neural networks in the first years has been stimulated by the introduction of the MNIST dataset comprising of 10-class handwritten digits [4]. Clanuwat et al. [105] recently introduced the Kuzushiji-MNIST a dataset focusing on Kuzushiji (cursive Japanese) that is clearly more challenging than the original MNIST because of the larger number of character classes.

Very ancient documents written on various supports are also attracting the attention of several researchers in the latest years. GRK-Papyri [11] is a dataset of greek handwriting on papyri used for testing writer identification algorithms. The dataset items are selected by experts from the field of Papyrology and consists of 50 handwriting samples in Greek on papyri approximately from the 6th century A.D., which have been produced by 10 different scribes. Palm leaves are another relevant type of support for writing in asian cultures. Lontar Sunda [8] is a dataset containing handwritten Sundanese palm leaf manuscripts from the 15th century containing 66 pages from 27 collections from Garut, West Java, Indonesia. The groundtruth information includes binarized images, annotations at word level and annotations at character level. The SleukRith dataset [9] contains Khmer Palm leaf manuscripts comprising 657 pages of Khmer palm leaf manuscripts randomly selected from different collections with variable quality. There are three types of data: isolated characters, words, and lines. The AMADI_LontarSet [7] contains handwritten Balinese palm leaf manuscripts. It includes binarized images groundtruth, word annotated images, and isolated characters. The dataset is built from 100 pages of randomly selected collections of palm leaf manuscripts from Bali, Indonesia.

Non textual information is also important in historical document recognition. The MUSCIMA++ dataset [106] can be used for research on Optical Music Recognition since it contains handwritten musical scores. The dataset consists of 140 handwritten pages with 91,254 annotated notation symbols and 82,247 explicitly marked relationships between symbol pairs.

### 5.2. Experimental Platforms

Together with the availability of large training sets in many domains and the access to relatively cheap computational power provided by GPUs the third component that fueled the increased use of deep learning architectures in several domains is the development of powerful open-source frameworks for training the models.

In the area of historical document recognition several approaches used standard frameworks like Pytorch [107] (e.g., in Reference [67]) Tensorflow [108] and Keras [109] (e.g., References [22,77]). In other cases DIAR-specific tools have been recently proposed in order to provide to researchers easily accessible and efficient tools for handling the different steps required in the development of effective applications.

eScriptorium [110] is an open source platform for historical document analysis and annotation. It allows interested users to upload document collections, transcribe and segment them manually or automatically with the help of an OCR engine. HistCorp [111] is a platform for the distribution in a uniform, standardised format corpora (gathered from historical corpora) and other useful resources and tools. It is designed for the distribution of historical texts from various time periods and genres for several European languages. HInDoLA [17] is an unified cloud-based platform for annotation, visualization and machine learning-based layout analysis of historical manuscripts. It features a suitable annotation GUI, a graphical analytics dashboard and interfaces with some machine-learning based modules.

One important ingredient in the development of effective machine learning applications is the possibility to measure in a suitable way the performance of the proposed solution. An open evaluation tool for layout analysis is presented in Reference [112]. The tool standardizes the evaluation of layout analysis tasks at pixel level. It is both available as a standalone Java application and as a RESTful web service.

### 5.3. Competitions

As mentioned at the end of the previous section evaluating in a standardized way document recognition techniques is essential for measuring the progress of research in historical document recognition. Competitions organized during main conferences in the field have been instrumental both for the collection of benchmark datasets and for the design of measures of performance.

One of the first competitions on historical document recognition has been organized by Antonacopoulos et al. during the 2nd International Workshop on Historical Document Imaging and Processing (HIP2013) held together ICDAR 2013 with the task of performing layout analysis on newspapers (HNLA 2013) [113]. During ICDAR 2015 the ANDAR-TL (ANcestry Document Analysis and Recognition Text Lines) competition on text line detection in historical documents has been organized by Ancestry. The competition uses the ANDAR-TL-IK dataset of 1300 images consisting of text lines in paragraph form drawn from collections from the eighteenth and nineteenth century. The images are labeled with the position of the first character of the left-most word on each text line. The objective of the competition is to detect the locations of text line origin points in the images from a test set. One well known competition for performing layout analysis for challenging medieval manuscripts has been organized during ICDAR 2017—the DIVA-HisDB [114]. The organizers provided a dataset for pixel-labelling and text line segmentation. The dataset comprises medieval manuscripts with complex layout—the images contain many interlinear and marginal glosses as well as texts in various sizes and decorated letters

With the latest explosion of applications of deep learning for document image analysis discussed in Section 1 it is not surprising that several competitions have been organized during ICDAR 2019. The ICDAR 2019 Competition on Image Retrieval for Historical Handwritten Documents [19] investigates the performance of large-scale retrieval of historical document images based on writing style. The datasets provided by cultural heritage institutions and digital libraries, contain a total of 20,000 document images in three main classes (manuscripts, letters, charters and legal documents) made by about 10,000 writers. The ICDAR 2019 Competition on Historical Book Analysis (HBA2019) [115] aims at evaluating pixel-labeling methods using the HBA 1.0 dataset for historical book analysis. The competition provides a large experimental corpus and an evaluation protocol to allow a fair performance benchmarking of different methods. Two nested challenges are evaluated: challenge 1 evaluates how image analysis methods could discriminate the textual content from the graphical one; challenge 2 assesses the capabilities of pixel-labeling methods to separate the textual content according to different text fonts (e.g., lowercase, uppercase, italic, etc.). By looking at non-latin scripts the ICDAR 2019 Historical Document Reading Challenge on Large Structured Chinese Family Records [24] proposes a large database of Chinese family records. The historical document reading challenge aims at analyzing an recognizing the layout, and finally detect and recognize the text lines and characters of the large collection containing more than 10,000 pages.

From the previous brief summary of recent competitions on the analysis of historical documents it is once again clear that the number of documents available for training and testing deep learning based methods significantly increased in the last years.

### 5.4. Synthetic Data Generation

Data augmentation is nowadays considered as one of the most effective techniques when dealing with small or unbalanced datasets. The technique consists on synthetically increasing the amount of available data, so that it can be used for training deep learning models which will most likely benefit from this process.

As just mentioned, data augmentation implies the existence and usage of synthetically generated data. As the name suggests, a synthetic dataset contains data that is generated with specific software tools. Such data is therefore not collected by any real-life survey or experiment: its main purpose is to be flexible and rich enough to increase and improve deep learning models performances.

This strategy has been considered also for historical document recognition, mainly when large training sets are not available because of the rarity of such documents. One solution proposed in several domains is the synthetic generation of data via deep learning methods itself, as detailed below. Martínek et al. [93] propose the synthetic training data generation for OCR or handwritten text recognition, similarly to Gaur et al. [116]. Author’s contribution is two-sided: at first, they collect a small dataset of isolated characters from historical document images and then build historical looking text lines from the generated characters. Such characters are generated using varying writing styles of the required language and processed to appear similar to ancient handwritings. Secondly, the authors designed and implemented an OCR system based on a convolutional LSTM network.

Semi-synthetic data augmentation for scanned historical documents is discussed in Reference [72]. The proposed method extracts background-only images and text-only images from different sources and mixes them in order to create semi-synthetic images. The approach improves the performance of a semantic segmentation and baseline extraction tool when tested on a public and a private datasets.

The generation of learning samples for historical handwriting recognition was studied and implemented with the use of image degradation techniques in Reference [117]. Other approaches addressed the synthesis of historical documents in various application fields: Pondenkandath et al. [84] with the goal of transforming modern printed document images into historical handwritten ones, giving to the results the “historical style”, Journet et al. [118] with the goal of circumventing the need of manually annotating real documents proposed the generation of massive ground-truthed data with high variability. Similar techniques are proposed in Reference [73] and described in Reference [119].

## 6. Final Remarks

The deep learning revolution touched several research fields and the analysis and recognition of historical documents is not an exception. A large number of papers have been published on this topic in the last few years with a significant increase in the number of tasks addressed and techniques considered. While previous research on DIAR for historical documents mostly focused on text recognition and keyword spotting, recent applications cover novel topics ranging from manuscripts dating to text location in maps. Several works now deal with the identification of individual text lines, while previously layout analysis was more focused on the segmentation of regions in mostly printed documents.

By looking at this research from a methodological point of view (the perspective of artificial neural networks) various deep learning methods are nowadays adopted, with a particular emphasis on architectures based on convolutional layers. In particular, several models are designed to produce an output having the same size of the input image thus providing a pixel labeling used in other contexts for semantic segmentation. Before the introduction of CNNs, pixel labeling was made by means of a sliding window feeding the neural network at different positions. On the opposite, the use of convolutional layers allows to obtain the desired pixel labeling by training the network on whole pages or large images patches.

Similarly to other research fields, the research on historical document analysis and recognition by means of deep learning architectures has been possible because of the large amount of labeled training data that is now available. Recent research datasets are not only larger, but they also cover novel types of documents and gather items from different areas of the world, not limiting the concept of cultural heritage to the western culture. Different problems also stimulated the design and test of innovative architectures or the re-use of models initially proposed for other tasks.

The tasks addressed by the methods analyzed in this paper are so diverse that it is difficult to make a meaningful comparison of results achieved because different data and different measures of performance are proposed when describing techniques proposed to address similar tasks. In general, methods based on deep learning succeed, in almost all the analyzed cases, in approaching the state of the art or even improving its performance and feasibility. The greatest difficulty, still encountered, is certainly that of finding a quantity of varied, consistent, and sufficient data to train models based on deep learning. One category of tasks with significant improvements are various techniques proposed for layout analysis and in particular for text line segmentation and baseline detection. The improved results have been achieved thanks to the availability of larger annotated datasets and to the use of connectionist architectures aimed at a semantic segmentation of input images. Today, deep learning seems to be the panacea for several research areas including historical document processing. However, two important factors that might slow down the widespread adoption of these techniques in the area are the need for large training data and the reduced explainability of models obtained by deep learning methods. These factors can be problematic for the acceptance and adoption of deep learning techniques by scholars in the Humanities that are the final users of these tools.

Even if from one point of view the diversity of tasks addressed is a positive aspect of the research on historical document processing, since it comes out from different research needs in the Humanities, from another perspective this is a limit because research datasets are fragmented and focused on different topics. Identifying a grand-challenge—a topic of interest for a broad community—could be beneficial for pushing the research in the area, similarly to what happened in other fields like object detection in natural images and scene text detection and recognition. Without such goal the researchers will continue to work with relatively small datasets (even if larger than few years ago) and would not be able to design and evaluate novel architectures proposed. The analysis of historical documents is definitely one of the research fields in which the lack of data, the absence of high-quality data, the scarcity of extensive and complete datasets are on the agenda, precisely because of the particular nature of the documents analyzed. The composition of such documents, often dated back to ancient times and composed by ancient materials, ancestors of paper, increases the complexity of handing down and digitizing certain documents, which is the main object of study in this work. Robust and scalable techniques for data augmentation and synthetic document generation, specifically designed for historical documents, seem to be the most needed tools that could further push the research in the area.

In addition to the previous challenges and the need for synthetic document generation, we can foresee some directions for future research in the area. Despite the fact that several applications still deal with text-only documents, historical documents often contain also graphical information and numerical values organized in tabular structures. Recognizing the information in these types of documents is a promising direction for new research where architectures designed in other research fields can be suitably used. One example is the use of algorithms and methods, originally designed for scene text detection and recognition, to perform keyword spotting in graphical documents or in works difficult to read. Other architectures, developed for object detection in natural images, and successfully applied for floor plan recognition, can be adapted to address the recognition of graphical historical documents, for the identification of particular illuminated letters or, for instance, stamps or other distinctive marks that can help in the identification of previous owners or producers of the works. Another emerging sub-field of research in DIAR is related to the understanding of tabular information in contemporary documents (including digital-born documents like PDFs), one task that is still far from being solved in an unconstrained way. Very recent advances in this research can be suitably used also to address the recognition of archival documents organized in tables, like census records.

## Figures and Tables

**Table 1:** Distribution of papers using neural network based approaches published in the proceedings of the International Conference of Document Analysis and Recognition (ICDAR) conference series from 1991 to 2019.

	1991	1993	1995	1997	1999	2001	2003	2005	2007	2009	2011	2013	2015	2017	2019
ANN Papers	4	11	17	11	9	7	8	6	3	4	5	7	35	63	195
ANN (%)	3.8	4.2	6.5	4.8	4.4	2.9	3.3	2.3	1.2	1.3	1.4	2.7	14.6	29.8	85.5
Text (%)	100	64	65	73	33	43	37	66	33	75	100	86	80	41	35

**Table 2:** Most frequent combinations of input and output types and their applications.

Input	Output	Papers
	Page	[46,72,73,74]
	Region	[56,75,76]
	Table	[25]
Page	Text line	[14,52,53,54,58]
		[20,57,77,78]
	Bounding box	[27,40]
	Transcribed text	[13,18,70,79]
		[60,63,80]
	Numerical value	[26,28]
Image patch	Image patch	[12,22,42,44]
	Numerical value	[15]
Region	Transcribed text	[68,81,82,83]
Text	Image	[84]

**Table 3:** Features of main datasets used in historical document image analysis.

Dataset	Task	Type of Doc.	Type of GT	Number of Items	Ref.
George	Keyword	Handwritings	Word-level	20 pages	[61]
Washington	Spotting		annotations	656 text lines	
Esposalles	Handwriting	Archival	Word-level	173 pages,	[62]
	Recognition	Documents	annotations	1747 registers	
				and 5447 lines	
READ-BAD	Baseline	Archival	Page-level	2036 pages	[23]
	Detection	Documents	annotations		
DIVA-HisDB	Layout	Medieval	Page Layout	150 pages	[100]
	Analysis	Manuscripts		3 manuscripts	
St.Gall	Handwriting	Handwritten	Word and line	60 pages	[101]
	Rec./Layout	Latin Manuscripts	level annotations	1410 text-lines	
	Analysis			11,597 words	
Parzival	Handwriting	Handwritten	Word and line	47 pages	[102]
	Rec./Layout	German Manuscripts	level annotations	4477 text-lines	
	Analysis			23,478 words	
VML-HD	Character	Arabic	Character and	680 pages	[103]
	Recognition	Scripts	sub-words level	1731 sub-words	
			bounding boxes	1731 sub-words	
Pinkas	Page	Hebrew	Word, line and	30 pages	[104]
Dataset	Segmentation	Manuscripts	page-level annotations	13,744 words	
Kuzushiji	Character	Kuzushiji	Character-level	49 character	[105]
-MNIST		Characters	annotations	classes	
GRK-Papyri	Writer	Handwritten	Writer	50 pages	[11]
	Identification	Papyri	id	10 writers	
Lontar	Binarization,	Sudanese palm	Binarized images,	66 pages	[8]
Sunda	Recognition	leaf Manuscripts	word and character	27 collections	
			level annotations		
SleukRith	Binarization,	Khmer palm	Character, word and	657 pages	[9]
Sunda	Recognition	leaf Manuscripts	line-level annotations		
AMADI	Binarization	Balinese palm	Binarized images,	100 pages	[7]
_LontarSet		leaf manuscripts	word and character		
			level annotations		
Muscima++	Music	Handwritten	Notation	140 pages	[106]
	Recognition	music Pages	graph	91,254 symbols	
